# Di-μ-chlorido-bis­{[2-(8-quinol­yloxy)­acetato-κ^3^ *N*,*O* ^1^,*O* ^2^]copper(II)}

**DOI:** 10.1107/S1600536808031656

**Published:** 2008-10-18

**Authors:** Zhi-hong Wang, Jun Fan, Wei-guang Zhang, Jun Wang

**Affiliations:** a School of Chemistry and Environment, South China Normal University, Guangzhou 510006, People’s Republic of China

## Abstract

The title compound, [Cu_2_(C_11_H_8_NO_3_)_2_Cl_2_], is a bicopper(II) complex. Each Cu^II^ ion is five-coordinated by two O atoms and one N atom from the (8-quinol­yloxy)acetate ligand, and by two μ_2_-chloride ligands, thus exhibiting a distorted square-pyramidal CuCl_2_NO_2_ coordination environment. Each (8-quinol­yloxy)acetate anion acts as a tridentate chelating ligand. In the crystal structure, adjacent quinolyl rings are involved in strong π–π stacking inter­actions, with inter­planar distances of 3.549 (5) and 3.763 (5) Å, thereby forming a two-dimensional planar network perpendicular to the *ab* plane. Furthermore, a weak inter­action [2.750 (4) Å] is observed within these planes between one Cu^II^ ion and a carboxyl­ate O atom from a ligand in an adjacent mol­ecule, which also contributes to the stability of the structure.

## Related literature

For general background, see: Hong *et al.* (2006 ▶); Sudik *et al.* (2005 ▶); Dong *et al.* (2007 ▶); Tong *et al.*, 1999 ▶. For related structures, see: Wang & Lu (2004 ▶); Wang *et al.* (2005 ▶). Koelsch (1931 ▶) reports the synthesis of the (8-quinol­yloxy)acetate ligand.
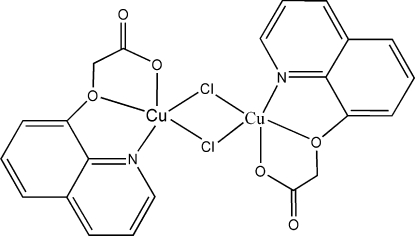


## Experimental

### 

#### Crystal data

[Cu_2_(C_11_H_8_NO_3_)_2_Cl_2_]
*M* *_r_* = 602.35
Monoclinic, 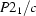

*a* = 8.3796 (17) Å
*b* = 19.195 (4) Å
*c* = 13.392 (3) Å
β = 98.85 (3)°
*V* = 2128.4 (8) Å^3^
*Z* = 4
Mo *K*α radiation
μ = 2.29 mm^−1^
*T* = 298 (2) K
0.36 × 0.30 × 0.24 mm

#### Data collection

Bruker SMART APEXII CCD area-detector diffractometer
Absorption correction: multi-scan (*SADABS*; Sheldrick, 1996 ▶) *T*_min_ = 0.462, *T*_max_ = 0.582
11625 measured reflections
4179 independent reflections
2737 reflections with *I* > 2σ(*I*)
*R*_int_ = 0.049

#### Refinement

*R*[*F* ^2^ > 2σ(*F* ^2^)] = 0.040
*wR*(*F* ^2^) = 0.087
*S* = 1.01
4179 reflections
307 parameters
H-atom parameters constrained
Δρ_max_ = 0.44 e Å^−3^
Δρ_min_ = −0.47 e Å^−3^

### 

Data collection: *APEX2* (Bruker, 2004 ▶); cell refinement: *SAINT* (Bruker, 1999 ▶); data reduction: *SAINT*; program(s) used to solve structure: *SHELXS97* (Sheldrick, 2008 ▶); program(s) used to refine structure: *SHELXL97* (Sheldrick, 2008 ▶); molecular graphics: *SHELXTL* (Sheldrick, 2008 ▶) and *ORTEP-3* (Farrugia, 1997 ▶); software used to prepare material for publication: *SHELXTL*.

## Supplementary Material

Crystal structure: contains datablocks I, global. DOI: 10.1107/S1600536808031656/zl2140sup1.cif

Structure factors: contains datablocks I. DOI: 10.1107/S1600536808031656/zl2140Isup2.hkl

Additional supplementary materials:  crystallographic information; 3D view; checkCIF report

## Figures and Tables

**Fig. 1.**
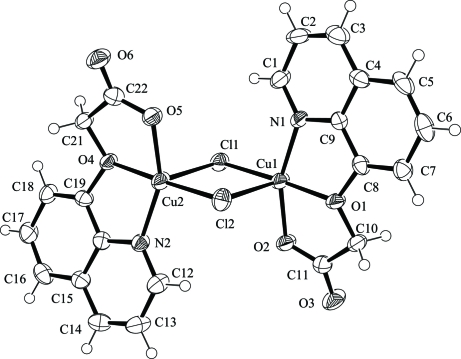
An ORTEP-3 (Farrugia, 1997) plot of (I), with displacement ellipsoids at the 50% probability level. All H atoms are drawn as spheres of arbitrary radius.

**Fig. 2.**
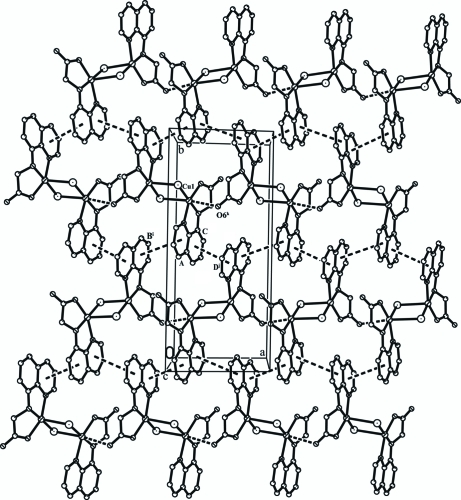
The packing diagram of (I), viewed perpendicular to the ab plane, showing the two-dimensional planar network generated by the π-π and weak Cu···O interactions. All H atoms have been omitted for clarity. [Symmetry codes: (i): -x, 1/2 + y, 1/2 - z; (j): 1 - x, 1/2 + y, 1/2 - z].

## supplementary crystallographic information

### Comment

Metal–polycarboxylate coordination polymers have attracted considerable attention in past decades, owing to their fascinating architectures and potential applications as new materials in gas absorption, catalysis and luminescence (Dong *et al.*, 2007; Sudik *et al.*, 2005). However, only a limited amount of work has been reported on the use of benzene polycarboxylate ligands that combine characteristics of both flexibility and rigidity (Hong *et al.*, 2006; Wang & Lu, 2004; Wang *et al.*, 2005).

Herein, we report the crystal structure of the title compound, (I). A perspective view of the binuclear copper complex (I), showing the atomic numbering scheme, is depicted in Fig. 1. The coordination geometry around the Cu^II^ ion may be described as a slightly distorted square pyramid, the basal plane being defined by one N atom, two O atoms from (8-quinolyloxy)acetate and one chloride anion; the apical position is occupied by another bridging chloride anion from the adjacent copper(II) unit, this atom being coordinated at a longer distance [Cu1—Cl2 = 2.823 (14) Å and Cu2—Cl1 = 2.776 (12) Å]. Thus, two chlorides form bi-bridges between two Cu^II^ ions, which link two Cu^II^ units to generate a binuclear complex. Each (8-quinolyloxy)acetate molecule acts as a tridentate chelating ligand. The inequivalence of the carboxylate C—O distances may be correlated with their involvement in bonding with the Cu^II^ centres.

In the crystal structure, adjacent quinolinyl rings are involved in strong π-π stacking attractions by partial overlapping of π-electron densities (Tong *et al.*, 1999). The centroid-centroid separation between rings A (atoms N1/C1—C4/C9) and B^i^ [atoms C15—C20; symmetry code: (i): - *x*, 1/2 + *y*, 1/2 - *z*] is 3.763 (5) Å, and the other between rings C (atoms C4—C9) and D*^j^* [atoms N2/C12—C15/C20; symmetry code: (*j*): 1- *x*, 1/2 + *y*, 1/2 - *z*] is 3.549 (5) Å. Considering these π-π intermolecular attractions, they imply the formation of a two-dimensional planar network perpendicular to the *ab* plane (Fig. 2). Furthermore, a weak interaction [2.750 (4) Å] is observed between the atom Cu1 and carboxylate oxygen atom O6^k^ [symmetry code: (k): -1+x,y,z] from the ligand in adjacent Cu^II^ unit, thus contributing to the two-dimensional network's stability (Fig. 2).

### Experimental

The ligand quinolin-8-yloxyacetic acid was prepared according to the general procedure reported by Koelsch (1931). An aqueous solution of CuCl_2_.2 H_2_O (0.057 g, 0.33 mmol) was added dropwise to the mixture of Y(NO_3_)_3_.6 H_2_O (0.064 g, 0.17 mmol) and quinolin-8-yloxyacetic acid (0.103 g, 0.50 mmol) in aqueous solution at 343 K, and the pH value was adjusted to be about 5 with NaOH. After stirring for 0.5 h, the resulting green solution was filtered. Slow evaporation from the filtrate for several weeks yielded green block-like crystals suitable for X-ray analysis. IR (KBr pellet, cm^-1^): 3051, 1650, 1628, 1505, 1429, 1379, 1317, 1263, 1115, 837, 772.

### Refinement

All the H atoms were placed in calculated positions and were allowed to ride on their parent atoms; C—H = 0.93 (aromatic C—H) and 0.97 (methylene) Å and *U*_iso_(H) = 1.2 *U*_eq_ of the carrier atom.

### Crystal data

**Table:**

[Cu_2_(C_11_H_8_NO_3_)_2_Cl_2_]	*F*(000) = 1208
*M**_r_* = 602.35	*D*_x_ = 1.880 Mg m^−^^3^
Monoclinic, *P*2_1_/*c*	Mo *K*α radiation, λ = 0.71073 Å
Hall symbol: -P 2ybc	Cell parameters from 2969 reflections
*a* = 8.3796 (17) Å	θ = 1.9–27.8°
*b* = 19.195 (4) Å	µ = 2.30 mm^−^^1^
*c* = 13.392 (3) Å	*T* = 298 K
β = 98.85 (3)°	Block, green
*V* = 2128.4 (8) Å^3^	0.36 × 0.30 × 0.24 mm
*Z* = 4	

### Data collection

**Table:**

Bruker SMART APEXII CCD area-detector diffractometer	4179 independent reflections
Radiation source: fine-focus sealed tube	2737 reflections with *I* > 2σ(*I*)
graphite	*R*_int_ = 0.049
φ and ω scans	θ_max_ = 26.0°, θ_min_ = 1.9°
Absorption correction: multi-scan (SADABS; Sheldrick, 1996)	*h* = −9→10
*T*_min_ = 0.462, *T*_max_ = 0.582	*k* = −19→23
11625 measured reflections	*l* = −16→13

### Refinement

**Table:**

Refinement on *F*^2^	Primary atom site location: structure-invariant direct methods
Least-squares matrix: full	Secondary atom site location: difference Fourier map
*R*[*F*^2^ > 2σ(*F*^2^)] = 0.040	Hydrogen site location: inferred from neighbouring sites
*wR*(*F*^2^) = 0.087	H-atom parameters constrained
*S* = 1.01	*w* = 1/[σ^2^(*F*_o_^2^) + (0.0256*P*)^2^ + 1.1172*P*] where *P* = (*F*_o_^2^ + 2*F*_c_^2^)/3
4179 reflections	(Δ/σ)_max_ = 0.001
307 parameters	Δρ_max_ = 0.44 e Å^−^^3^
0 restraints	Δρ_min_ = −0.47 e Å^−^^3^

### Special details

**Table:**

Geometry. All esds (except the esd in the dihedral angle between two l.s. planes) are estimated using the full covariance matrix. The cell esds are taken into account individually in the estimation of esds in distances, angles and torsion angles; correlations between esds in cell parameters are only used when they are defined by crystal symmetry. An approximate (isotropic) treatment of cell esds is used for estimating esds involving l.s. planes.
Refinement. Refinement of F^2^ against ALL reflections. The weighted R-factor wR and goodness of fit S are based on F^2^, conventional R-factors R are based on F, with F set to zero for negative F^2^. The threshold expression of F^2^ > 2sigma(F^2^) is used only for calculating R-factors(gt) etc. and is not relevant to the choice of reflections for refinement. R-factors based on F^2^ are statistically about twice as large as those based on F, and R- factors based on ALL data will be even larger.

### Fractional atomic coordinates and isotropic or equivalent isotropic displacement parameters (Å^2^)

**Table:**

	*x*	*y*	*z*	*U*_iso_*/*U*_eq_	
C1	0.8714 (5)	−0.0068 (2)	0.8758 (3)	0.0368 (10)	
H1	0.8366	0.0265	0.9181	0.044*	
C2	0.8505 (5)	−0.0778 (2)	0.8966 (3)	0.0432 (12)	
H2	0.8054	−0.0908	0.9531	0.052*	
C3	0.8959 (5)	−0.1275 (2)	0.8345 (3)	0.0428 (12)	
H3	0.8799	−0.1744	0.8473	0.051*	
C4	0.9673 (5)	−0.10730 (19)	0.7508 (3)	0.0325 (10)	
C5	1.0242 (5)	−0.1536 (2)	0.6825 (4)	0.0409 (12)	
H5	1.0099	−0.2012	0.6900	0.049*	
C6	1.0990 (5)	−0.1304 (2)	0.6066 (4)	0.0434 (12)	
H6	1.1356	−0.1624	0.5631	0.052*	
C7	1.1226 (5)	−0.05832 (19)	0.5921 (3)	0.0345 (10)	
H7	1.1760	−0.0427	0.5403	0.041*	
C8	1.0658 (5)	−0.01268 (18)	0.6549 (3)	0.0282 (9)	
C9	0.9887 (4)	−0.03519 (19)	0.7356 (3)	0.0270 (9)	
C10	1.1952 (5)	0.09524 (18)	0.6061 (3)	0.0322 (10)	
H10A	1.1737	0.0926	0.5329	0.039*	
H10B	1.3003	0.0748	0.6291	0.039*	
C11	1.1912 (5)	0.17117 (19)	0.6411 (3)	0.0321 (10)	
C12	0.6792 (5)	0.2987 (2)	0.6160 (3)	0.0381 (11)	
H12	0.7235	0.2651	0.5784	0.046*	
C13	0.6801 (5)	0.3683 (2)	0.5850 (3)	0.0437 (12)	
H13	0.7255	0.3802	0.5282	0.052*	
C14	0.6146 (5)	0.4184 (2)	0.6376 (3)	0.0404 (11)	
H14	0.6133	0.4646	0.6163	0.049*	
C15	0.5487 (5)	0.40025 (19)	0.7247 (3)	0.0302 (10)	
C16	0.4806 (5)	0.4478 (2)	0.7871 (3)	0.0371 (11)	
H16	0.4763	0.4950	0.7709	0.044*	
C17	0.4217 (5)	0.4254 (2)	0.8700 (3)	0.0380 (11)	
H17	0.3793	0.4578	0.9105	0.046*	
C18	0.4230 (5)	0.35414 (19)	0.8967 (3)	0.0340 (10)	
H18	0.3814	0.3392	0.9536	0.041*	
C19	0.4869 (5)	0.30816 (18)	0.8366 (3)	0.0288 (9)	
C20	0.5517 (4)	0.32890 (18)	0.7511 (3)	0.0273 (9)	
C21	0.3712 (5)	0.20080 (18)	0.8940 (3)	0.0307 (10)	
H21A	0.2681	0.2219	0.8677	0.037*	
H21B	0.3863	0.2032	0.9672	0.037*	
C22	0.3754 (5)	0.1251 (2)	0.8592 (3)	0.0323 (10)	
Cl1	0.88233 (13)	0.17203 (5)	0.87627 (8)	0.0355 (3)	
Cl2	0.68751 (13)	0.12051 (5)	0.62854 (8)	0.0378 (3)	
Cu1	0.98473 (6)	0.11091 (2)	0.75955 (4)	0.03462 (16)	
Cu2	0.59143 (6)	0.18353 (2)	0.74655 (4)	0.03365 (16)	
N1	0.9383 (4)	0.01413 (15)	0.7986 (2)	0.0284 (8)	
N2	0.6180 (4)	0.27902 (15)	0.6966 (2)	0.0284 (8)	
O1	1.0725 (3)	0.05920 (12)	0.6493 (2)	0.0345 (7)	
O2	1.0939 (3)	0.18630 (12)	0.7016 (2)	0.0350 (7)	
O3	1.2868 (4)	0.21096 (14)	0.6113 (2)	0.0504 (9)	
O4	0.5008 (3)	0.23677 (12)	0.85579 (19)	0.0296 (6)	
O5	0.4801 (3)	0.10898 (12)	0.8035 (2)	0.0369 (7)	
O6	0.2728 (4)	0.08555 (14)	0.8829 (2)	0.0501 (8)	

### Atomic displacement parameters (Å^2^)

**Table:**

	*U*^11^	*U*^22^	*U*^33^	*U*^12^	*U*^13^	*U*^23^
C1	0.037 (3)	0.038 (2)	0.040 (3)	−0.0059 (19)	0.018 (2)	−0.001 (2)
C2	0.047 (3)	0.042 (3)	0.043 (3)	−0.010 (2)	0.017 (2)	0.015 (2)
C3	0.045 (3)	0.028 (2)	0.055 (3)	−0.005 (2)	0.006 (2)	0.007 (2)
C4	0.031 (3)	0.025 (2)	0.040 (3)	−0.0021 (17)	0.001 (2)	0.0057 (19)
C5	0.042 (3)	0.017 (2)	0.062 (3)	0.0001 (18)	0.002 (2)	−0.002 (2)
C6	0.048 (3)	0.031 (2)	0.049 (3)	0.009 (2)	0.001 (2)	−0.015 (2)
C7	0.039 (3)	0.032 (2)	0.032 (3)	0.0041 (18)	0.006 (2)	−0.0038 (19)
C8	0.029 (2)	0.021 (2)	0.034 (2)	0.0002 (16)	0.0036 (19)	−0.0039 (17)
C9	0.025 (2)	0.026 (2)	0.030 (2)	0.0031 (16)	0.0046 (18)	0.0019 (18)
C10	0.035 (3)	0.033 (2)	0.032 (2)	−0.0030 (18)	0.0151 (19)	0.0014 (19)
C11	0.034 (3)	0.029 (2)	0.034 (3)	−0.0018 (18)	0.008 (2)	0.0014 (19)
C12	0.042 (3)	0.039 (2)	0.036 (3)	−0.007 (2)	0.015 (2)	−0.004 (2)
C13	0.054 (3)	0.043 (3)	0.037 (3)	−0.009 (2)	0.016 (2)	0.006 (2)
C14	0.048 (3)	0.031 (2)	0.043 (3)	−0.006 (2)	0.009 (2)	0.008 (2)
C15	0.028 (2)	0.028 (2)	0.033 (3)	−0.0051 (17)	0.0011 (19)	0.0008 (18)
C16	0.040 (3)	0.022 (2)	0.047 (3)	0.0005 (18)	0.000 (2)	−0.004 (2)
C17	0.042 (3)	0.027 (2)	0.048 (3)	0.0005 (19)	0.014 (2)	−0.009 (2)
C18	0.038 (3)	0.030 (2)	0.037 (3)	−0.0006 (18)	0.017 (2)	−0.0072 (19)
C19	0.031 (2)	0.023 (2)	0.032 (2)	−0.0030 (17)	0.0058 (19)	0.0014 (18)
C20	0.028 (2)	0.022 (2)	0.032 (2)	−0.0033 (16)	0.0042 (18)	−0.0042 (17)
C21	0.032 (2)	0.028 (2)	0.036 (3)	−0.0038 (17)	0.0158 (19)	−0.0004 (18)
C22	0.038 (3)	0.029 (2)	0.030 (3)	−0.0011 (18)	0.005 (2)	0.0003 (18)
Cl1	0.0459 (7)	0.0303 (5)	0.0342 (6)	0.0017 (4)	0.0184 (5)	−0.0031 (4)
Cl2	0.0448 (7)	0.0367 (6)	0.0346 (6)	0.0054 (5)	0.0142 (5)	−0.0073 (5)
Cu1	0.0460 (3)	0.0222 (3)	0.0414 (3)	−0.0020 (2)	0.0248 (2)	−0.0008 (2)
Cu2	0.0420 (3)	0.0234 (3)	0.0405 (3)	−0.0009 (2)	0.0220 (2)	−0.0033 (2)
N1	0.0268 (19)	0.0278 (17)	0.033 (2)	−0.0023 (14)	0.0128 (15)	0.0011 (15)
N2	0.030 (2)	0.0251 (17)	0.032 (2)	−0.0029 (14)	0.0101 (16)	−0.0027 (15)
O1	0.0459 (19)	0.0225 (14)	0.0404 (18)	−0.0029 (12)	0.0241 (14)	0.0024 (12)
O2	0.0457 (19)	0.0259 (14)	0.0383 (18)	−0.0044 (13)	0.0220 (14)	−0.0003 (13)
O3	0.055 (2)	0.0372 (17)	0.067 (2)	−0.0154 (15)	0.0353 (18)	−0.0038 (16)
O4	0.0359 (17)	0.0216 (13)	0.0355 (17)	−0.0016 (11)	0.0192 (13)	−0.0022 (12)
O5	0.0466 (19)	0.0231 (14)	0.0464 (19)	−0.0008 (13)	0.0240 (15)	−0.0021 (13)
O6	0.055 (2)	0.0334 (16)	0.070 (2)	−0.0102 (15)	0.0363 (18)	−0.0018 (16)

### Geometric parameters (Å, °)

**Table:**

C1—N1	1.312 (5)	C14—C15	1.409 (6)
C1—C2	1.409 (5)	C14—H14	0.9300
C1—H1	0.9300	C15—C20	1.414 (5)
C2—C3	1.357 (6)	C15—C16	1.416 (5)
C2—H2	0.9300	C16—C17	1.353 (6)
C3—C4	1.405 (6)	C16—H16	0.9300
C3—H3	0.9300	C17—C18	1.414 (5)
C4—C5	1.408 (6)	C17—H17	0.9300
C4—C9	1.414 (5)	C18—C19	1.359 (5)
C5—C6	1.349 (6)	C18—H18	0.9300
C5—H5	0.9300	C19—O4	1.396 (4)
C6—C7	1.416 (5)	C19—C20	1.398 (5)
C6—H6	0.9300	C20—N2	1.372 (5)
C7—C8	1.351 (5)	C21—O4	1.445 (4)
C7—H7	0.9300	C21—C22	1.528 (5)
C8—O1	1.383 (4)	C21—H21A	0.9700
C8—C9	1.409 (5)	C21—H21B	0.9700
C9—N1	1.377 (5)	C22—O6	1.224 (5)
C10—O1	1.433 (4)	C22—O5	1.274 (5)
C10—C11	1.533 (5)	Cl1—Cu1	2.2290 (12)
C10—H10A	0.9700	Cl2—Cu2	2.2362 (12)
C10—H10B	0.9700	Cu1—O2	1.937 (3)
C11—O3	1.218 (5)	Cu1—N1	1.985 (3)
C11—O2	1.269 (5)	Cu1—O1	2.011 (3)
C12—N2	1.320 (5)	Cu2—O5	1.929 (3)
C12—C13	1.400 (5)	Cu2—N2	1.976 (3)
C12—H12	0.9300	Cu2—O4	2.026 (3)
C13—C14	1.357 (6)	Cu1—Cl2	2.8232 (14)
C13—H13	0.9300	Cu2—Cl1	2.7761 (12)
			
N1—C1—C2	122.2 (4)	C17—C16—H16	119.6
N1—C1—H1	118.9	C15—C16—H16	119.6
C2—C1—H1	118.9	C16—C17—C18	121.7 (4)
C3—C2—C1	120.2 (4)	C16—C17—H17	119.1
C3—C2—H2	119.9	C18—C17—H17	119.1
C1—C2—H2	119.9	C19—C18—C17	117.8 (4)
C2—C3—C4	119.4 (4)	C19—C18—H18	121.1
C2—C3—H3	120.3	C17—C18—H18	121.1
C4—C3—H3	120.3	C18—C19—O4	123.9 (4)
C3—C4—C5	124.9 (4)	C18—C19—C20	122.6 (4)
C3—C4—C9	117.6 (4)	O4—C19—C20	113.5 (3)
C5—C4—C9	117.4 (4)	N2—C20—C19	118.5 (3)
C6—C5—C4	121.6 (4)	N2—C20—C15	122.4 (4)
C6—C5—H5	119.2	C19—C20—C15	119.1 (4)
C4—C5—H5	119.2	O4—C21—C22	107.0 (3)
C5—C6—C7	121.1 (4)	O4—C21—H21A	110.3
C5—C6—H6	119.5	C22—C21—H21A	110.3
C7—C6—H6	119.5	O4—C21—H21B	110.3
C8—C7—C6	118.6 (4)	C22—C21—H21B	110.3
C8—C7—H7	120.7	H21A—C21—H21B	108.6
C6—C7—H7	120.7	O6—C22—O5	125.1 (4)
C7—C8—O1	126.2 (4)	O6—C22—C21	117.4 (4)
C7—C8—C9	121.7 (3)	O5—C22—C21	117.3 (3)
O1—C8—C9	112.0 (3)	O2—Cu1—N1	158.25 (12)
N1—C9—C8	118.6 (3)	O2—Cu1—O1	79.98 (11)
N1—C9—C4	121.8 (4)	N1—Cu1—O1	80.80 (12)
C8—C9—C4	119.6 (4)	O2—Cu1—Cl1	98.29 (9)
O1—C10—C11	106.5 (3)	N1—Cu1—Cl1	101.28 (10)
O1—C10—H10A	110.4	O1—Cu1—Cl1	177.22 (8)
C11—C10—H10A	110.4	O5—Cu2—N2	155.37 (13)
O1—C10—H10B	110.4	O5—Cu2—O4	80.24 (11)
C11—C10—H10B	110.4	N2—Cu2—O4	81.42 (12)
H10A—C10—H10B	108.6	O5—Cu2—Cl2	97.29 (9)
O3—C11—O2	125.9 (4)	N2—Cu2—Cl2	101.01 (10)
O3—C11—C10	116.7 (4)	O4—Cu2—Cl2	177.52 (8)
O2—C11—C10	117.3 (3)	C1—N1—C9	118.8 (3)
N2—C12—C13	122.4 (4)	C1—N1—Cu1	128.2 (3)
N2—C12—H12	118.8	C9—N1—Cu1	113.0 (2)
C13—C12—H12	118.8	C12—N2—C20	118.4 (3)
C14—C13—C12	120.1 (4)	C12—N2—Cu2	128.4 (3)
C14—C13—H13	119.9	C20—N2—Cu2	113.0 (3)
C12—C13—H13	119.9	C8—O1—C10	122.8 (3)
C13—C14—C15	119.7 (4)	C8—O1—Cu1	115.4 (2)
C13—C14—H14	120.2	C10—O1—Cu1	115.1 (2)
C15—C14—H14	120.2	C11—O2—Cu1	118.3 (2)
C14—C15—C20	117.0 (4)	C19—O4—C21	119.2 (3)
C14—C15—C16	125.0 (4)	C19—O4—Cu2	113.1 (2)
C20—C15—C16	118.0 (4)	C21—O4—Cu2	113.6 (2)
C17—C16—C15	120.7 (4)	C22—O5—Cu2	118.1 (2)
			
N1—C1—C2—C3	−1.9 (7)	O1—Cu1—N1—C1	179.2 (4)
C1—C2—C3—C4	1.5 (7)	Cl1—Cu1—N1—C1	−2.7 (4)
C2—C3—C4—C5	177.9 (4)	O2—Cu1—N1—C9	−27.2 (5)
C2—C3—C4—C9	0.1 (6)	O1—Cu1—N1—C9	0.9 (3)
C3—C4—C5—C6	−176.6 (4)	Cl1—Cu1—N1—C9	179.0 (2)
C9—C4—C5—C6	1.2 (6)	C13—C12—N2—C20	0.6 (6)
C4—C5—C6—C7	−0.4 (7)	C13—C12—N2—Cu2	175.1 (3)
C5—C6—C7—C8	−1.2 (6)	C19—C20—N2—C12	−179.9 (4)
C6—C7—C8—O1	−177.3 (4)	C15—C20—N2—C12	−0.8 (6)
C6—C7—C8—C9	1.9 (6)	C19—C20—N2—Cu2	4.7 (4)
C7—C8—C9—N1	178.0 (3)	C15—C20—N2—Cu2	−176.2 (3)
O1—C8—C9—N1	−2.7 (5)	O5—Cu2—N2—C12	−138.4 (4)
C7—C8—C9—C4	−1.0 (6)	O4—Cu2—N2—C12	179.4 (4)
O1—C8—C9—C4	178.2 (3)	Cl2—Cu2—N2—C12	−1.1 (4)
C3—C4—C9—N1	−1.6 (6)	O5—Cu2—N2—C20	36.4 (5)
C5—C4—C9—N1	−179.5 (3)	O4—Cu2—N2—C20	−5.8 (2)
C3—C4—C9—C8	177.4 (4)	Cl2—Cu2—N2—C20	173.7 (2)
C5—C4—C9—C8	−0.5 (6)	C7—C8—O1—C10	−27.5 (6)
O1—C10—C11—O3	−179.3 (4)	C9—C8—O1—C10	153.3 (3)
O1—C10—C11—O2	3.5 (5)	C7—C8—O1—Cu1	−177.4 (3)
N2—C12—C13—C14	−0.8 (7)	C9—C8—O1—Cu1	3.4 (4)
C12—C13—C14—C15	1.2 (7)	C11—C10—O1—C8	−163.9 (3)
C13—C14—C15—C20	−1.4 (6)	C11—C10—O1—Cu1	−13.9 (4)
C13—C14—C15—C16	178.5 (4)	O2—Cu1—O1—C8	167.3 (3)
C14—C15—C16—C17	−179.3 (4)	N1—Cu1—O1—C8	−2.5 (2)
C20—C15—C16—C17	0.6 (6)	O2—Cu1—O1—C10	15.1 (2)
C15—C16—C17—C18	−1.1 (6)	N1—Cu1—O1—C10	−154.7 (3)
C16—C17—C18—C19	0.5 (6)	O3—C11—O2—Cu1	−167.7 (3)
C17—C18—C19—O4	177.8 (3)	C10—C11—O2—Cu1	9.2 (5)
C17—C18—C19—C20	0.6 (6)	N1—Cu1—O2—C11	14.9 (5)
C18—C19—C20—N2	178.1 (4)	O1—Cu1—O2—C11	−13.4 (3)
O4—C19—C20—N2	0.6 (5)	Cl1—Cu1—O2—C11	168.8 (3)
C18—C19—C20—C15	−1.0 (6)	C18—C19—O4—C21	39.6 (5)
O4—C19—C20—C15	−178.5 (3)	C20—C19—O4—C21	−143.0 (3)
C14—C15—C20—N2	1.3 (6)	C18—C19—O4—Cu2	177.1 (3)
C16—C15—C20—N2	−178.7 (3)	C20—C19—O4—Cu2	−5.4 (4)
C14—C15—C20—C19	−179.6 (4)	C22—C21—O4—C19	152.5 (3)
C16—C15—C20—C19	0.4 (5)	C22—C21—O4—Cu2	15.1 (4)
O4—C21—C22—O6	−179.0 (3)	O5—Cu2—O4—C19	−157.3 (2)
O4—C21—C22—O5	−2.6 (5)	N2—Cu2—O4—C19	6.2 (2)
C2—C1—N1—C9	0.5 (6)	O5—Cu2—O4—C21	−17.3 (2)
C2—C1—N1—Cu1	−177.8 (3)	N2—Cu2—O4—C21	146.2 (3)
C8—C9—N1—C1	−177.7 (3)	O6—C22—O5—Cu2	163.8 (3)
C4—C9—N1—C1	1.3 (6)	C21—C22—O5—Cu2	−12.3 (5)
C8—C9—N1—Cu1	0.7 (4)	N2—Cu2—O5—C22	−25.9 (5)
C4—C9—N1—Cu1	179.7 (3)	O4—Cu2—O5—C22	16.5 (3)
O2—Cu1—N1—C1	151.1 (3)	Cl2—Cu2—O5—C22	−163.8 (3)
